# The Recreovía of Bogotá, a Community-Based Physical Activity Program to Promote Physical Activity among Women: Baseline Results of the Natural Experiment Al Ritmo de las Comunidades

**DOI:** 10.3390/ijerph14060633

**Published:** 2017-06-13

**Authors:** Olga L. Sarmiento, Ana Paola Rios, Diana C. Paez, Karoll Quijano, Rogério César Fermino

**Affiliations:** 1 Department of Public Health, School of Medicine, Universidad de los Andes, Carrera 1, nº 18A-12. Centro, Bogotá CEP 111711, DC, Colombia; a-rios@uniandes.edu.co (A.P.R.); paez.d.carolina@gmail.com (D.C.P.); karoll.quijano@gmail.com (K.Q.); 2 Postgraduate Program in Physical Education, Federal University of Technology—Brazil, Pedro Gusso St, 2.601, Neoville, Curitiba CEP 81310-900, PR, Brazil; rogeriofermino@hotmail.com; 3 Research Group of Physical Activity and Quality of Life (GPAQ), Pontifical Catholic University of Parana, Imaculada Conceição St, 1.155, Prado Velho, Curitiba CEP 80215-901, PR, Brazil

**Keywords:** physical activity, parks, dancing

## Abstract

Community-based physical activity (PA) programs in Latin America have been recognized because of the use of available environmental resources to offer PA classes. Yet, the evaluation of programs focused on PA classes involving dancing in public spaces is limited. The aim of this study was to assess the physical activity levels, park use, and the contextual characteristics of public parks with and without the Recreovía in Bogotá in Colombia. Al Ritmo de las Comunidades is a natural experiment conducted in nine parks (3 parks implementing new Recreovías, 3 control parks and 3 parks with existing Recreovías) during 2013. We used the System for Observing Play and Recreation in Communities to evaluate park use (gender, age, and physical activity level) and target areas. A total of 4925 people were observed during 702 observation visits to parks. The percentage of women was higher in parks with Recreovía, compared to parks without Recreovía (53% vs. 40% vs. 33%; *p* < 0.001). Women using parks with Recreovía compared to women in parks without Recreovía were less likely to be sedentary (25% vs. 39%; *p* < 0.0001) and more likely to engage in moderate-to-vigorous activity (75% vs. 61%; *p* < 0.0001). Among men, the activity pattern was the opposite. The Recreovía is a promising strategy to promote park use and PA, especially among women who are less likely to meet PA recommendations during their leisure time. The provision of a cost-free community program may be an effective approach and a good investment for health.

## 1. Introduction

Currently, nearly a quarter of the world’s adult population is physically inactive [1]. The biggest burden falls on low- and middle-income countries (LMICs), where non-communicable diseases (NCDs) are increasing at the highest rate [1,2]. A total of 28 million deaths from NCDs occur in LMICs [2]. Specifically, in Latin America, interventions face the challenges of the rapid growth of cities, urbanization, access to motorization, widespread inequality, lack of space, and limited recreational resources, including parks [3]. Thus, activity-friendly urban design could be an effective strategy to promote physical activity (PA) [4]. Specifically, parks may influence health by shaping behaviors [1,4,5] and have been identified as important environmental resources for promoting leisure-time PA [4,6]. However, numerous studies showed that most park areas were vacant and that park users were largely sedentary [6]. Furthermore, men are more likely to be active than women [3,7,8,9,10]. In addition, most studies have been conducted in high-income countries [10], but data from Latin American countries are limited to a few studies conducted in Brazil [7,11].

Within parks, organized park programs may increase PA [8]. Specifically, free and regular programs of PA classes in parks have emerged as a promising and scalable strategy for promoting PA at the community level, especially for women [1,12]. Currently, physical activity classes in parks and public spaces are implemented worldwide [12]. A large proportion of these programs is in Latin American countries (LACs), in which 45% of the countries have implemented this type of program [12]. These programs comprise free PA classes of rumba, folklore, aerobics, flexibility, and martial arts. The health benefits of these types of classes, of which dancing is a main component, include promotion of PA and increased quality of life related to health [13,14].

Latin American countries such as Brazil, Ecuador, and Colombia are settings of exemplary community-based PA programs to increase PA and promote the use of existing physical resources and combine them with PA classes and health counseling [12,15]. Specifically, the evaluation of the program Academia de Cidade in Brazil (ACP) showed that parks with ACP were visited more, particularly by women and older adults [7,11,16]. In addition, people using parks with existing PA classes or living near ACP were more likely to engage in moderate-to-vigorous PA [14,16,17].

**Figure 1:**
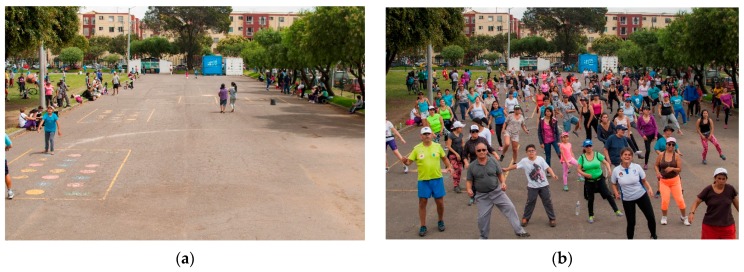
(**a**) Target area in a park without the Recreovía program and; (**b**) The same park target area with the Recreovía program. The park is located in a low-income neighborhood. Photo by Karoll Quijano.

**Figure 2:**
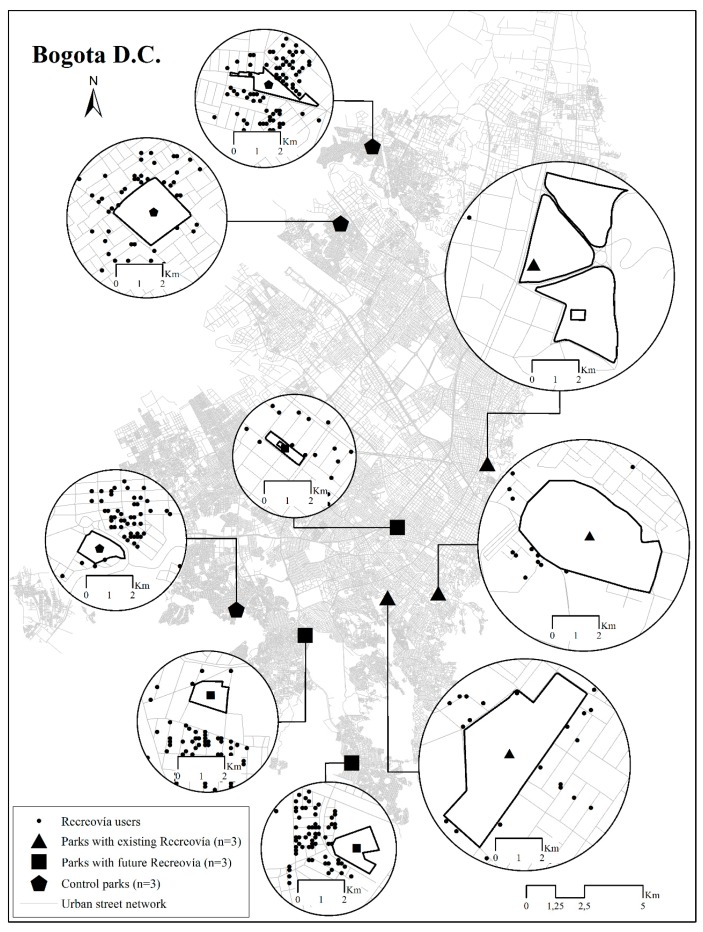
Parks of Bogotá, by presence of Recreovía Program (*n* = 9). The black dots represent households of the participants.

In Bogotá, Colombia, the Recreovía community program promotes PA classes in a safe and inclusive manner, contributing to the adoption of healthy lifestyles among users [12] (Figure 1a,b and Figure 2). The Recreovía program started in 1995 as a program to complement the Ciclovía program, to increase the use of parks in the city and to provide free recreational activities for vulnerable socioeconomic populations [12,18]. The Recreovía program offers free-of-charge PA classes led by trained instructors on weekdays, Sundays, and holidays. The classes include aerobics with a cultural dancing component (e.g., salsa, rumba, merengue, reggaeton, folk dance, etc.), strength, stretching, and classes for children. The program is currently implemented in 41 settings in the city. Five are located in metropolitan parks, 23 in zonal parks, one in a neighborhood park, one in a community center, 10 in shopping centers, and 1 in a prison [12]. In seven parks, the PA classes are conducted only on weekdays from 6:30 to 8:30 am and from 6:30 to 8:30 pm. In 10 parks, the classes are conducted on Sundays and holidays from 8:00 am to 1:00 pm. In 12 parks, the PA classes are conducted on weekdays and on Sundays. The Recreovía program has high coverage of the city, reaching 19 out of 20 administrative localities [12].

Understanding the role of the Recreovía program in shaping active communities is limited and requires a contextual analysis. We hypothesize that females, who are more likely to enjoy dancing, will be more likely to be seen engaging in moderate-to-vigorous PA in parks with Recreovía compared to parks without Recreovía. Therefore, the aim of this study was to assess the physical activity levels, park use, and the contextual characteristics of public parks with and without the Recreovía program in Bogotá, Colombia.

## 2. Materials and Methods

### 2.1. Study Setting

Bogotá is the capital of Colombia, with 7.8 million people [19]. The city has an average temperature of 14 °C with alternate periods of rain and drought rather than seasons. Socioeconomic status (SES) in Bogotá is determined by using the classification from the city Planning Department, which has six categories based on physical characteristics of the household and neighborhood area. SES category 1 corresponds to the poorest and category 6 to the richest.

Currently, Bogotá is recognized for its efforts to promote PA by providing access to recreational and active community facilities [20] such as the community-based PA programs Ciclovía [21] and Recreovía in 29 parks [12]. The total area of parks in Bogota is 29,440,838 m^2^ within an urban area of 384,000,000 m^2^ [22]. There are 5050 parks distributed in the city that are classified as follows: 1 regional park (area: 2,670,000 m^2^), 33 metropolitan parks (area: >100,000 m^2^), 78 zonal parks (area: 1000 m^2^–100,000 m^2^), 3317 neighborhood parks (>1000 m^2^), and 1601 pocket parks (<1000 m^2^). In addition, 20 parks are in the process of evaluation and categorizing according to their area by the District Institute of Sports and Recreation of Bogotá (IDRD in Spanish: *Instituto Distrital de Recreación y Deporte*) [23].

### 2.2. Study Design

This study is part of the project Al Ritmo de las Comunidades, a natural experiment to determine the effectiveness of Recreovía at increasing PA levels at the community level in Bogota, Colombia [14]. In the year 2013, the director of the IDRD implemented three new Recreovía programs in zonal parks which provided a unique opportunity for a natural experiment. This natural experiment incorporated a traditional control group (parks without Recreovía) and a naturally occurring comparison group (existing Recreovía) as a post-intervention control to account for outcomes that are the result of 20 years of implementation of a real community program. This paper describes a contextual evaluation of the baseline results of this study. Data collection was conducted between September and November of 2013. The project was approved by the ethics committee of the Universidad de los Andes (Act 161 of 2012).

The study was conducted in nine public parks. Parks were classified into three groups. Group 1 included parks implementing future Recreovía programs on Sundays (*n* = 3 zonal parks), selected by the coordinator of the program based on community requests and the SES of the neighborhood. Group 2 included control parks (*n* = 3 zonal parks) without Recreovía. The control parks were selected randomly from a list of parks previously matched to the characteristics of the parks in Group 1. First, parks were matched by type of park (neighborhood versus zonal); second, parks were matched by the neighborhood SES (low [1–2 strata] versus middle [3–4 strata]; third, parks were matched by the availability of five types of PA target areas (yes versus no). PA target areas included courts, fields, walking/running tracks, exercise areas, open areas, and Recreovía areas. Recreovía target areas corresponded to courts or plazas. Open areas corresponded to grass areas adapted for PA and recreation which are common in Brazil and Colombia [7]. Group 3 consisted of parks with existing Recreovía programs of at least 12 years’ duration (*n* = 3 metropolitan parks). To select the parks from Group 3, we used the list of existing Recreovía hubs in parks, provided by the program coordinator of the IDRD, which was sorted by attendance during the year 2012. From this list, one park was selected randomly from the top 5% of highest attendance and one park was selected randomly from the lowest 5% attendance. The third park corresponded to the oldest Recreovía hub that started in 1995 and represented 20 years of program implementation (Figure 2).

### 2.3. Measurement of Physical Activity

#### 2.3.1. System for Observing Play and Recreation in Communities (SOPARC)

We used the System for Observing Play and Recreation in Communities (SOPARC) to assess the gender, age group, and PA level of park users [24,25]. SOPARC was developed to obtain observational data on the number of participants and their PA levels during PA and leisure activities in public places [24]. SOPARC is based on systematic observations (scans) of selected areas (target areas) from left to right (approximately 1 s per person) while using a mechanical counter to register the data. SOPARC has been widely used to evaluate parks and public spaces in the community and has been used recently in Latin America [7,11]. For our study, we used a SOPARC method adapted from the Brazilian method, which had simply been translated into Spanish [11]. The observation protocol and forms were translated by a native Spanish speaker, fluent in Portuguese, and checked for accuracy.

#### 2.3.2. Evaluation of Target Areas

Target areas were pre-selected areas of parks, characterized by their allowance of the practice of PA (e.g., Recreovía, courts, fields, walking/running tacks, exercise areas, open areas). A Recreovía target area corresponded to courts or plazas (Figure 2). We created maps for each park, delineating target areas that were evaluated and coded according to the type of area, the presence of structures for PA practices (lines, soccer goal-posts, etc.), and the type of surface (grass, concrete, etc.). The number of target areas in parks ranged from 11 to 45. Parks with an existing Recreovía program had between 11 and 45 target areas. Parks implementing future Recreovía programs had 16 to 25 target areas. Control parks had 17 to 22 target areas.

#### 2.3.3. Observation of Park Target Areas and Park Users

In each park, observations were made during one Sunday between September and October 2013. Other days of the week were not included because the selected parks only run the program on Sundays.

On each observation day, in the Recreovía target areas and the potential Recreovía target areas of parks without the program, three observation periods were conducted for about 20 min each at 8:00 am, 10:00 am, and 12:00 pm. In addition, in the parks with existing Recreovía programs, four observation periods during PA classes were conducted for about 15 min each, at 10 min before the class, 10 min after the beginning of the class, 40 min after the beginning of the class, and 10 min after ending the class. The three classes observed began at 8:00 am, 10:00 am and 12:00 pm. In non-Recreovía target areas of parks, two observation periods were conducted for about 20 min each at 8:00 am and 3:00 pm.

In all parks, the total data were collected during 702 observation visits. The number of observation visits in each park ranged from 44 to 110. In parks with an existing Recreovía program, the number of observation visits ranged from 44 to 167. In parks implementing future Recreovía programs, the number of observation visits ranged from 54 to 86. In control parks, the number of observation visits ranged from 47 to 64.

Data were collected by two trained observers under the supervision of two field coordinators. Following the SOPARC observation protocol, we scored each person observed in the area by gender, age group (child 0–12 years, adolescents 13–20 years, adults 21–59, or older adults ≥60 years), and PA level, which included sedentary (lying down, sitting, or standing), moderate (e.g., walking slowly, moving) and vigorous (e.g., walking fast, running, aerobic classes, playing soccer). The observations were performed first for women and subsequently for men. In addition, the contextual characteristics of target areas (i.e., accessible, practically equipped, supervised, activity organized, dark, empty) were assessed and rated as yes/no through the observations [5].

### 2.4. Training of Observers

The observers were trained by researchers who are experts in the use of SOPARC (Rogerio César Fermino and Ana Paola Rios). The training included theoretical and practical components. The theoretical component was conducted by an expert during a 1-day workshop (6 h), in which trained observers were familiarized with the SOPARC method (operational definitions, instruments notation, coding conventions, and categorization of the PA levels and age groups by gender). In addition, instructions for registering and completing the SOPARC form using a mechanical counter were also given. SOPARC training materials, available at the Active Living Research website [26], were used to train the observers. The practical component was conducted over three days (seven hours) of field work in parks, comparing results between observers and receiving feedback from the coordinators.

To simulate the real conditions of observations and to obtain a minimum agreement to start the data collection, six target areas (1 walking/running trail, 1 open area, 2 exercise areas, and 2 courts for sports) were selected from a park that was not included in the study. In total, 118 observations, 3022 individuals (1827 men and 1195 women) were observed with an average of 26 users per observation. The percentage of agreement was calculated according to the protocol’s recommendations and the results were analyzed by gender, age group, and PA level with an intraclass correlation coefficient (ICC) SPSS (v. 17.0; SPSS Inc., Chicago, IL, USA). For women, the agreement for age group and PA levels was 73% and 66%, respectively (ICC: 0.995 and 0.994; *p*-value < 0.001). For men, age and PA showed agreements of 78% and 77%, respectively (ICC: 0.996 and 0.982; *p*-value < 0.001). These results are consistent with other studies conducted in Brazil and the United States [7,8,9,11,27].

### 2.5. Physical Activity Resource Assessment of Parks

The Physical Activity Resource Assessment (PARA) instrument was developed to systematically document and describe the type, features, amenities, and quality of PA resources in urban neighborhoods [28,29]. This instrument is easy to use, has a high reliability (% agreement > 81%) [30], and was translated and adapted to the Latin American context to evaluate the quality of parks by trained researchers in Brazil and Colombia.

The PARA identifies and qualifies the conditions of parks into five domains: (1) features for PA practices (fields and courts for sports, exercise areas, trails for walking, running, skating, roller-skating, swimming pools, playgrounds); (2) amenities (bathrooms, benches, locker rooms, lighting, trash cans, picnic tables); (3) incivilities (cleanliness, aesthetics, safety, dog refuse, garbage, broken glass, graffiti/tagging, vandalism, overgrown grass, unrestrained dogs, litter); (4) services (restaurants, libraries, PA materials, PA classes); and (5) accessibility (taxi and bus stops, parking, bike racks, and bicycle paths).

The features and amenities of the parks are classified into four categories: “not present” (code: 0), “poor” (code: 1), “mediocre” (code: 2), and “good” (code: 3). Incivilities are classified as “not present” (code: 3), “small presence” (code: 2), “average presence” (code: 1), or “very present” (code: 0). Services and accessibility items are classified as “absence” (code: 0) and “presence” (code: 1). The quality score was computed as the sum of the items described above.

### 2.6. Geographic Information System Data

We estimated the percentage of park users adjusted by population density within 500 m from the park boundaries and by age (children 0–9 years; adolescents 10–19 years, adults 20–59 years, and older adults ≥60 years). This percentage is defined by the following formula:

$$P\left(park\;users\right)=\frac{\#\;park\;users\;observed\;by\;age\;group}{\#\;\mathrm{inhabitants}\;\mathrm{within}\;500\;m\;of\;the\;park\;boundaries\;by\;age\;group}\times 100$$

where *#* of park users by age group were measured by SOPARC and *#* of inhabitants within 500 m of the park boundaries were obtained from the census [31]. The information was calculated using ArcGIS 9.0 (ESRI, Inc., Redlands, CA, USA).

### 2.7. Data Analysis

To describe the park and user characteristics for each group, we used descriptive statistics (absolute and relative frequency distribution, mean, standard deviation, and range). We first described the characteristics of the parks and target areas (quantity, size, and quality). We then described user characteristics (gender, age and PA levels). All comparisons between categorical variables were tested with a chi-squared test (*χ*^2^), and comparisons of continuous variables were tested by one-way ANOVA or the Kruskal–Wallis test. Multilevel multiple regression models were conducted to examine the associations of the independent variables (intervention group, park type, and the neighborhood SES) with the outcome measurements (overall number of park visitors, number of visitors being sedentary, number of visitors being moderately to vigorously active). All analyses were performed using SPSS (v. 17.0, SPSS Inc., Chicago, IL, USA) and STATA (ver. 14.0, StataCorp LP: College Station, TX, USA).

## 3. Results

### 3.1. Characteristics of Parks and Target Areas

The average park size was 47,801 ± 50,829 m^2^. A total of 210 target areas were observed (average size 353.9 ± 577.1 m^2^) (Table 1 and Figure 2). Parks with existing Recreovía programs were larger (average size 113,634 ± 20,020 m^2^) and had more target areas (*n* = 95), compared with parks implementing future Recreovía programs (average size 14,547 ± 7083 m^2^ and target areas *n* = 60) and control parks (average size 15,223 ± 11,474 m^2^ and target areas *n* = 55). However, this difference was only marginally statistically significant (*p*-value = 0.066) (Table 1). In all parks, sports areas were the target area most frequently observed (51%), while the exercise/stretching areas were less likely to be observed (3.8%, *p*-value < 0.001) (Table 1). Parks with existing Recreovía programs showed more sports areas (63.2%) than parks implementing future Recreovía programs (41.7%) and control parks (40.0%) (*p*-value < 0.001). Control parks showed more open areas (21.8%) than parks with existing Recreovía programs (0%) and parks implementing future Recreovía programs (10%) (*p*-value < 0.001) (Table 1). Almost all target areas in the three types of park were usable (99.4%) and accessible (99.0%), and only 12.9% were supervised (Table 1).

### 3.2. Quality of Parks

The average scores of features, amenities, incivilities, and services and accessibility were 34.3 ± 18.0; 11.8 ± 5; 19.8 ± 7.6; 2.4 ± 1.3, and 2.3 ± 1.0, respectively. Although not statistically significant (*p*-value = 0.110), the parks with existing Recreovía had higher quality scores in all indicators (Table 2).

### 3.3. Park Users

We observed 4925 park users, from which 68.5% were observed in parks with existing Recreovía (Table 3). More women (53.0%) and adults (71.0%) were observed in parks with existing Recreovía compared with parks with future Recreovía (women: 39.6%, adults: 44.6%) and control parks (women: 32.6%, adults: 44.8%) parks (*p*-value < 0.001) (Table 3).

**Figure 3:**
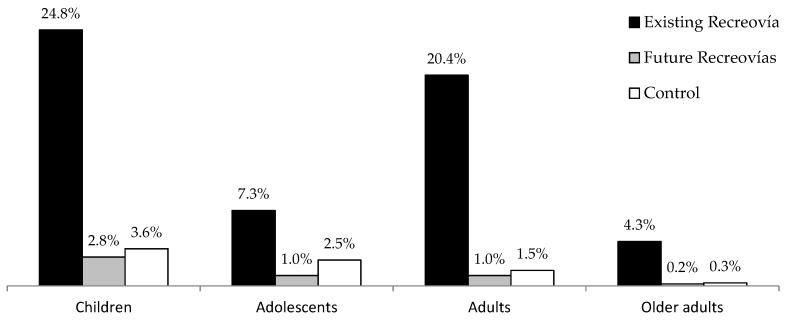
Percentage of park users adjusted by population density, represented by age group and presence of the Recreovía program.

The number and percentage of park users adjusted by population density of children, adolescents, adults and older adults was 18,204 (15.8%); 21,826 (19.7%); 64,337 (57.4%) and 8022 (7.3%), respectively. Parks with existing Recreovía were more used by people from all age groups compared to parks without Recreovía (*p*-value < 0.001) (Figure 3).

### 3.4. Activities Observed in Target Areas in Parks with and without the Recreovía Program

Overall, the main activities performed by women were aerobics (7.7%), walking (7.0%), and basketball (6.6%). The least common activities performed by women were swinging (0.6%) and running (0.5%) (Table 2). In parks with existing Recreovía, the main activity of women was aerobics (21.2%). In parks with future Recreovía, the main activity performed by women was skating (5.9%). In control parks, the main activity performed by women was basketball (11.4%). The parks implementing future Recreovía programs and control parks had higher percentages of empty areas when compared to the parks with existing Recreovía (*p*-value < 0.001). In 55.1% of all the target areas, women were not observed (Table 2).

Overall, the main activities performed by men were soccer (14.3%), basketball (10.1%), and standing up (8.5%). The least common activities performed by men were jogging/running and stretching (0.6%), swinging (0.5%), and skateboarding (0.4%) (Table 2). In parks with existing Recreovía, the main activity of men was aerobics (20.3%). In parks implementing future Recreovía programs, the main activity of men was soccer (13.7%). In control parks, the main activity of men was basketball (20.4%). The control parks had higher percentages of empty areas when compared to the parks with existing Recreovía programs and future Recreovía programs (*p*-value < 0.001). In 38.1% of all the target areas, men were not observed (Table 2).

### 3.5. PA Levels in Parks with and without a Recreovía Program

**Figure 4:**
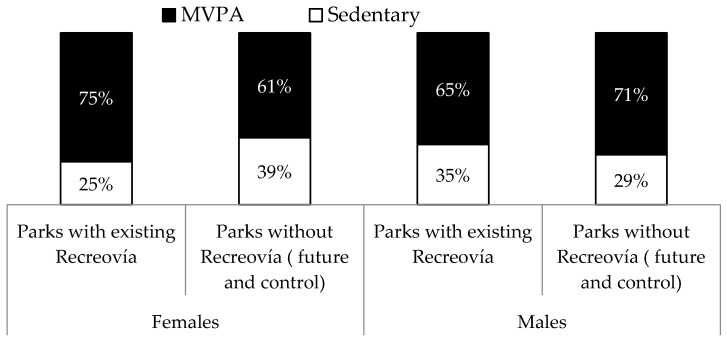
Physical activity intensity level (MVPA: moderate-to-vigorous physical activity) by gender and park program (existing Recreovía vs. no Recreovía program) in Bogotá, Colombia, 2013.

Women observed in parks with existing Recreovía were more likely to be engaged in moderate-to-vigorous physical activity (MVPA), compared with women observed in parks without Recreovía (future Recreovía and control) (75% versus 61%; *p*-value < 0.001) (Figure 4). Among men, the results showed the opposite pattern; they were more likely to be engaged in MVPA in parks without Recreovía, compared to men observed in parks with Recreovía (71% versus 65%, *p*-value < 0.01) (Figure 4).

### 3.6. Patterns of PA Levels in Parks before, during and after Recreovía Program Activities

**Figure 5:**
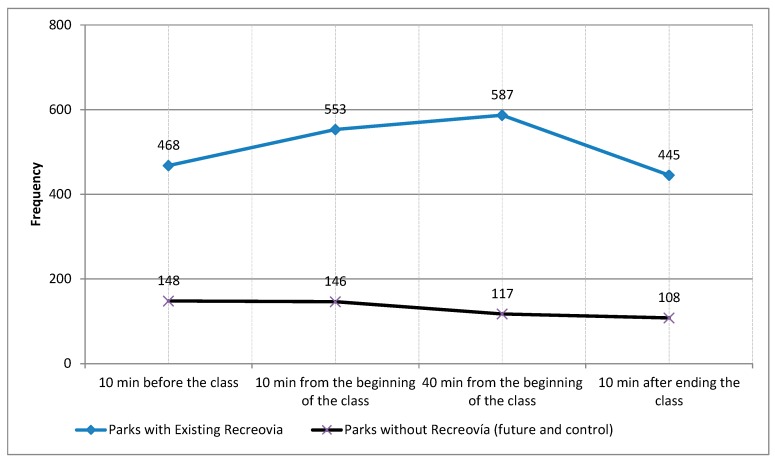
Number of participants observed in the potential target areas for aerobics in parks before, during and after the Recreovía park program (existing Recreovía vs. no Recreovía program) in Bogotá, Colombia, 2013.

When the target areas of PA classes in the parks with existing Recreovía were observed, we found an increase in the observed number of persons 10 min and 40 min after class started compared to the before and after class periods of time. In contrast, 10 min after the class had ended, we found a decrease in the observed number of persons (*p*-value < 0.001). Likewise, we observed more persons engaged in MVPA activities during the Recreovía class compared to the before and after class time periods (*p*-value < 0.001) (Figure 5).

## 4. Discussion

This study assessed the park use and the PA intensity level in public parks with and without the Recreovía program in Bogota, Colombia. Parks with Recreovía were more likely to be used by women. Parks with an existing Recreovía program had a higher percentage of users compared to the parks without the Recreovía program. The presence of the Recreovía program was associated with higher observed levels of MVPA among women. In contrast, the presence of the Recreovía program was associated with lower observed levels of MVPA among men. These results underline the importance of culturally-appropriate supervised activities such as PA classes to promote park use and MVPA among women in parks during the weekend.

Previous studies conducted in Brazil [11,17] and the US [32] showed that PA classes and supervised activities in public parks are associated with the higher use of parks by adults and higher PA levels. In the study conducted in Recife, more of the users of parks with Academia da Cidade (ACP) were females, compared to parks without ACP [11]. Similarly, in the study of a low-income, predominantly Latino neighborhood park in San Fernando, California, researchers found that the “100 Citizens” fitness program increased MVPA, mainly among women. The percentage of observed females in parks with ACP (45.1%) was lower than what we observed in the parks with the Recreovía program (53%). In contrast, the 100 Citizens fitness program had a higher percentage of observed women (55.6%). In addition, the percentage of the population engaging in MVPA in parks with ACP (63.7%) was lower than the observed percentage of persons engaged in MVPA in parks with Recreovía (70%). Additionally, cross-sectional studies conducted in Brazil [16,17] and Colombia [14] have shown that adults who participate in PA classes are more likely to meet PA recommendations. Together, these studies underline the importance of the provision of locally funded programs or free classes for increasing park use and the intensity levels of PA at the population level, especially for lower-income populations of Latin American origin.

Parks with Recreovía also showed high scores related to their features, amenities, incivilities, and services and accessibility indicators. Together, the quality score and the organized activities, such as the free PA classes, in part could explain the higher use of these parks [33]. Studies have shown that the quality of various attributes of a park, as well as the characteristics of its surroundings, are important predictors of the use of the site [34,35,36]. For example, in Curitiba (Brazil), the positive perception of the environment in the surrounding parks, and greater satisfaction with the sites, were positively associated with the use of urban parks [37,38]. Likewise, intervention studies with the implementation of new structures, renovations, and improvement of the quality of parks have shown satisfactory results, with increased frequency of use and PA level practiced in the parks [6,39,40,41].

One of the main activities observed in parks with the Recreovía program was aerobics, which in Colombia and Brazil mainly involved dancing. Specifically, a systematic review showed that programs that promote dancing, such as Zumba, which is very popular in Latin America, could be an effective type of physical activity to improve the aerobic capacity and cardiovascular outcomes of members of the population [42]. Additionally, small but positive effects of Zumba include improvements in body composition, muscular strength, balance, and quality of life [42]. Future studies aimed at evaluating physical activity classes should consider evaluation at the individual level, taking into account anthropometric parameters, body composition, metabolic profile, muscular fitness parameters, and aerobic performance.

Physical activity classes in parks and public spaces have emerged as a promising intervention for increasing population levels of PA and for decreasing health inequalities by reaching low-income women who are at higher risk of inactivity [12,16,18]. Since the year 2000, the implementation of PA classes in public spaces has increased significantly, as a reflection of practice-based evidence. Currently, these programs are implemented in at least 350 cities in Argentina, Brazil, Chile, Colombia, Cuba, Ecuador, Guatemala, and Mexico. The scalability of this type of multi-sectorial program in Colombia has been associated with the investment in the working conditions and training of instructors and the allocation of public funds. There has also been a request for accountability, a diversification of resources, a presence of community support and champions at different levels and positions, and continuous advocacy to include physical activity in public policies [12,43]. Future studies should be aimed at assessing the impact of this type of real-world intervention. These studies will require the design of natural experiments by transdisciplinary teams.

Some limitations should be considered when interpreting the results of this study. This paper describes cross-sectional baseline results of the project Al Ritmo de las Comunidades; the results of a natural experiment with Recreovía implementation will be presented in future publications. Thus, it is not possible to establish a causal relationship between the variables (quality of parks, presence of Recreovía and MVPA). Currently, we have an ongoing longitudinal, contextual evaluation with SOPARC of the three groups of parks that will be finished in the second semester of 2017. These ongoing results will provide relevant information of park use and PA after 4 years of implementation of the new Recreovía programs. A longitudinal analysis of a small sample of women who started attending the Recreovía classes showed a pattern indicating that MVPA minutes increased after 6 months of evaluation, but it was not statistically significant [14]. Since we only selected points where Recreovía was available in public parks, these results cannot be extrapolated to other Recreovía locations, such as in supermarkets and shopping and community centers. Our study could also be biased by confounding factors related to the characteristics of the built environment and social environment that cannot be determined by the data gathered. Nonetheless, parks were comparable to the SES of the neighborhoods in which they are located. Because SOPARC uses direct observation, observed participants could react to the presence of observers. However, observers reported that they were typically ignored during measurements. Despite the limitations of SOPARC with their large coverage and low cost, this methodology offers a useful tool for the assessment of PA. We are currently in the process of training personnel from IDRD in this tool so that they can continue the evaluation of PA levels with new parks implementing the Recreovía program.

## 5. Conclusions

Parks with Recreovía were more likely to be used by women and had a higher percentage of users compared to parks without the Recreovía program. The presence of the Recreovía program was also associated with higher observed levels of MVPA among women. Providing culturally-appropriate community PA and dancing classes in public parks during the weekends could be a promising strategy to promote PA among women.

## Figures and Tables

**Table 1:** Characteristics of parks and target areas, by presence of the Recreovía program in Bogotá, Colombia, 2013.

Variable	Parks with Existing Recreovías (*n* = 3)	Parks Implementing Future Recreovías (*n* = 3)	Control Parks (*n* = 3)	*p*	All Parks (*n* = 9)
Size of park (m^2^)					
Average ± SD	113,634 ± 20,020	14,547 ± 7083	15,223 ± 11,474	0.066 ^a^	47,801 ± 50,829
Minimum–maximum	96,257–135,527	8980–22,521	8182–28,464		8182–135,527
Characteristics of target areas					
Number	95	60	55	-	210
Size (m^2^)					
Average ± SD	437.9 ± 727.99	208.7 ± 108.4	355.9 ± 543.2	0.543 ^a^	353.9 ± 577.1
Minimum–maximum	50.0–3131	50.0–545.0	18.0–2883		18.0–3131
Type of area (%)					
Sports area	63.2	41.7	40.0	-	51.0
Playground area	13.7	16.7	12.7	-	14.3
Recreovía area	13.7	13.3 *	9.1 ^†^	-	12.4
Open area	0.0	10.0	21.8	-	8.6
Skating/roller track	4.2	3.3	10.9	-	5.7
Walking/running track	3.2	8.3	1.8	-	4.3
Strength/stretching exercise area	2.1	6.7	3.6	-	3.8
Conditions (%)					
Usable	99.7	100.0	98.2	-	99.4
Accessible	100.0	96.6	100.0	-	99.0
Equiped	50.8	23.5	32.9	-	38.4
Empty	19.0	52.9	19.8	-	29.2
Organized	42.1	1.0	9.6	-	22.1
Supervised	11.2	18.1	9.6	-	12.9

* Future Recreovía area; ^†^ potential Recreovía area; ^a^ Kruskal–Wallis.

**Table 2:** Characteristics of main activities observed in the parks by presence of the Recreovía program in Bogotá, Colombia, 2013.

Variable	Parks with Existing Recreovías (*n* = 3)	Parks Implementing Future Recreovías (*n* = 3)	Control Parks (*n* = 3)	*p*	All Parks (*n* = 9)
Main activity in the target area (%)					
Female (%)					
Not present	41.4	70.1	53.9	-	55.1
Aerobics (dance/step aerobics)	21.2	0.0	1.8	-	7.7
Walking	7.8	4.9	8.4	-	7.0
Basketball	3.4	4.9	11.4	-	6.6
Standing up	8.4	2.5	8.4	-	6.4
Skating	1.6	5.9	5.4	-	4.3
Sitting	5.9	4.9	1.8	-	4.2
Soccer	0.3	2.5	5.4	-	2.7
Yoga	4.1	0.0	0.0	-	1.4
Stretching	1.9	0.5	0.6	-	1.0
Strengthening exercises	2.5	0.0	0.0	-	0.8
Bicycling/BMX	0.0	2.0	0.0	-	0.7
Volleyball	0.9	0.5	0.6	-	0.7
Swinging	0.6	0.5	0.6	-	0.6
Jogging/running	0.0	1.0	0.6	-	0.5
Male (%)					
Not present	24.3	60.8	29.3	-	38.1
Soccer	9.4	13.7	19.8	-	14.3
Basketball	5.0	4.9	20.4	-	10.1
Standing up	10.9	4.9	9.6	-	8.5
Walking	6.9	7.8	9.0	-	7.9
Aerobics (dance/step aerobics)	20.3	0.0	0.0	-	6.8
Sitting	5.9	3.9	3.6	-	4.5
Bicycling/BMX	0.6	1.5	3.6	-	1.9
Yoga	4.1	0.0	0.0	-	1.4
Skating	2.8	0.5	0.6	-	1.3
Tennis/squash	3.4	0.0	0.0	-	1.1
Strengthening exercises	2.5	0.0	0.6	-	1.0
Volleyball	0.9	0.5	1.2	-	0.9
Jogging/running	0.3	1.0	0.6	-	0.6
Stretching	1.3	0.5	0.0	-	0.6
Swinging	0.3	0.0	1.2	-	0.5
Skateboard	1.3	0.0	0.0	-	0.4
Quality of park (score)					
Features for physical activity					
Average ± std. deviation	50.0 ± 25.2	27.7 ± 9.1	25.3 ± 4.9	0.505 ^a^	34.3 ± 18.0
Minimum–maximum	22.0–71.0	21.0–38.0	22.0–31.0		21.0–71.0
Amenities (score)					
Average ± std. deviation	14.3 ± 8.5	9.7 ± 1.2	11.3 ± 3.1	0.802 ^a^	11.8 ± 5.1
Minimum–maximum	8.0–24.0	8.0–12.0	8.0–14.0		8.0–24.0
Incivilities (score of cleaning, aesthetics and safety)					
Average ± std. deviation	17.3 ± 8.2	21.0 ± 8.7	21.0 ± 8.7	0.429 ^a^	19.8 ± 7.6
Minimum–maximum	8.0–23.0	11.0–27.0	11.0–26.0		8.0–27.0
Services (score)					
Average ± std. deviation	3.3 ± 1.5	2.3 ± 1.2	1.7 ± 0.6	0.264 ^a^	2.4 ± 1.3
Minimum–maximum	2.0–5.0	1.0–3.0	1.0–2.0		1.0–5.0
Accessibility (score)					
Average ± std. deviation	3.3 ± 1.2	2.0 ± 0.0	1.7 ± 0.6	0.110 ^a^	2.3 ± 1.0
Minimum–maximum	2.0–4.0	2.0–2.0	1.0–2.0		1.0–4.0
Population density within 500 m from the boundaries of the park ^‡^ N (Average ± std. deviation)					
Children (0–9 years)	2718 (906 ± 638)	6490 (2163 ± 1054)	8996 (2998 ± 1315)	0.121 ^b^	18,204 (2022 ± 1282)
	14.1%	16.4%	16.8%		15.8%
Adolescents (10–19 years)	3262 (1087 ± 652)	9034 (3011 ± 1930)	9530 (3176 ± 1388)	0.216 ^b^	21,826 (2425 ± 1591)
	16.9%	22.8%	17.8%		19.7%
Adults (20–59 years)	11,769 (3923 ± 1613)	21,327 (7109 ± 1814)	31,241 (10,413 ± 5651)	0.162 ^b^	64,337 (7148 ± 4166)
	61.0%	53.9%	58.4%		57.4%
Older adults (≥60 years)	1546 (515 ± 62)	2734 (911 ± 575)	3742 (1247 ± 805)	0.430 ^a^	8022 (891 ± 588)
	8.0%	6.9%	7.0%		7.3%

^‡^ Network buffer in 500 m; ^a^ Kruskal–Wallis; ^b^ ANOVA one way.

**Table 3:** Characteristics of park users by presence of the Recreovía program in Bogotá, Colombia, 2013 (*n* = 4925).

Variable	Parks with Existing Recreovías (*n* = 3)		Parks Implementing Future Recreovías (*n* = 3)		Control Parks (*n* = 3)		*p*	All Parks (*n* = 9)	
	*n*	%	*n*	%	*n*	%		*n*	%
Total users	3376	68.5	495	10.1	1054	21.4		4925	100.0
Sex									
Female	1790	53.0	196	39.6	344	32.6	<0.001	2330	47.3
Male	1586	47.0	299	60.4	710	67.4		2595	52.7
Age group									
Children	674	20.0	179	36.2	328	31.1	<0.001	1181	24.0
Adolescents	239	7.1	89	18.0	242	23.0		570	11.6
Adults	2397	71.0	221	44.6	472	44.8		3090	62.7
Older adults	66	2.0	6	1.2	12	1.1		84	1.7
Physical activity level									
General									
Sedentary	994	29.9	147	29.8	338	33.7	0.068	1479	30.7
Moderate	1808	54.3	255	51.6	520	51.8		2583	53.5
Vigorous	525	15.8	92	18.6	145	14.5		762	15.8
Female									
Sedentary	434	25.4	66	31.2	143	44.4	<0.001	643	28.6
Moderate	991	57.8	106	50.3	144	44.7		1241	55.3
Vigorous	287	16.8	39	18.5	35	10.9		361	16.1
Male									
Sedentary	560	34.7	81	28.6	195	28.6	0.022	836	32.4
Moderate	817	50.6	149	52.7	376	55.2		1342	52.0
Vigorous	238	14.7	53	18.7	110	16.2		401	15.6
